# CRISPR-Cas9 mediated targeted disruption of *FAD2–2 microsomal omega-6 desaturase* in soybean (*Glycine max.L*)

**DOI:** 10.1186/s12896-019-0501-2

**Published:** 2019-01-28

**Authors:** Noor al Amin, Naveed Ahmad, Nan Wu, Xiumin Pu, Tong Ma, Yeyao Du, Xiaoxue Bo, Nan Wang, Rahat Sharif, Piwu Wang

**Affiliations:** 1 College of Agronomy, Plant Biotechnology Center, Jilin Agricultural University, Changchun, 130118 Jilin China; 2 Ministry of Education Engineering Research Center of Bioreactor and Pharmaceutical, Development Jilin Agricultural University, Changchun, 130118 Jilin China

**Keywords:** CRISPR-Cas9, Targeted mutagenesis, FAD2–2, Oleic acid, Soybean

## Abstract

**Background:**

Recent innovation in the field of genome engineering encompasses numerous levels of plant genome engineering which attract the substantial excitement of plant biologist worldwide. RNA-guided CRISPR Cas9 system has appeared a promising tool in site-directed mutagenesis due to its innovative utilization in different branches of biology. CRISPR-Cas9 nuclease system have supersedes all previously existed strategies and their associated pitfalls encountered with site-specific mutagenesis.

**Results:**

Here we demonstrated an efficient sequence specific integration/mutation of FAD2–2 gene in soybean using CRISPR-Cas9 nuclease system. A single guided RNA sequence was designed with the help of a number of bioinformatics tools aimed to target distinct sites of FAD2–2 loci in soybean. The binary vector (pCas9-AtU6-sgRNA) has been successfully transformed into soybean cotyledon using Agrobacterium tumafacien. Taken together our findings complies soybean transgenic mutants subjected to targeted mutation were surprisingly detected in our target gene. Furthermore, the detection of Cas9 gene, BAR gene, and NOS terminator were carried out respectively. Southern blot analysis confirmed the stable transformation of Cas9 gene into soybean. Real time expression with qRT-PCR and Sanger sequencing analysis confirmed the efficient CRISPR-Cas9/sgRNA induced mutation within the target sequence of FAD2–2 loci. The integration of FAD2–2 target region in the form of substitution, deletions and insertions were achieved with notably high frequency and rare off-target mutagenesis.

**Conclusion:**

High frequent mutation efficiency was recorded as 21% out of all transgenic soybean plants subjected to targeted mutagenesis. Furthermore, Near-infrared spectroscopy (NIR) indicates the entire fatty acid profiling obtained from the mutants seeds of soybean. A considerable modulation in oleic acid content up to (65.58%) whereas the least level of linoleic acid is (16.08%) were recorded. Based on these finding CRISPR-Cas9 system can possibly sum up recent development and future challenges in producing agronomically important crops.

**Electronic supplementary material:**

The online version of this article (10.1186/s12896-019-0501-2) contains supplementary material, which is available to authorized users.

## Background

Genome editing is a critical and complex tool to study the functional genomics of plants in past decades. Amidst all previously adapted techniques such as virus-induced gene silencing, T-DNA/transposon insertion, antisense RNA, and RNA interference strategies CRISPR-Cas9 nuclease system have supersedes them all. Producing low-quality mutants, unwanted mutations, point mutation and several other pitfalls are associated with these classical methods. [1, 2]. These advances are good enough for carrying basic plant research however genetic crop improvement and constructing new mutant libraries require new tools for more precise genome editing, and regulation of gene expression. CRISPR-Cas9 system has remarkably captivated the title in 2011 as “methods of the year” in plant biology. This extensive system of type II clustered regularly interspaced short palindromic repeat CRISPR-associated protein 9 (Cas9) have been reported recently in a wide variety of plants including soybean [3], Arabidopsis [4], and rice [5, 6]. Underlying the principle of single nucleotide polymorphism, previously established ZFNs (Zinc Finger Nuclease) and TALENs (Transcription Activator-Like Effectors Nuclease) and CRISPR system paved the way for site directed mutagenesis. The advent of targeted mutation induced by CRISPR cas9 system has been proved very simple, efficient and predictable tool in functional genomics of many plants [7]. The genetic improvement of agronomical traits such as yield, Rearrangement, disease resistance, adaptation against various stresses and controlling negative regulation of specific genes via induced target mutagenesis are the potential areas of future researches [8, 9].

Soybean (*Glycine max* (L.) is an essential legume crop which provides a rich source of dietary proteins, animal feed, sustainable agricultural products, vegetable oil, and direct human consumption. Recent advances have expanded soybean research towards the production of biodiesel from the feedstock of soybean [10]. The utilization of soybean oils with high oleic acid content resulting in monounsaturated fatty acids could be useful in minimizing various health problems [11–13]. Researchers have been proved that Soybeans genome holds two similarly identical copies (*FAD2*–1 and *FAD2*–2) of microsomal 휔-6 desaturase [9]. The primary expression of *FAD2*–1 was confirmed during seed developmental processing unlike *FAD2*–2 which was expressing in vegetative parts as wells [10, 11]. The conserved sequence homology of the *FAD2*–2 family reveals multiple isoforms such as *FAD2*–1A, *FAD2–1B, FAD2*–2B and *FAD2*–2C which are nearly related to their predecessor family by sharing 99% homology in amino acid sequence [14–16]. By these means, this confirmed the importance and pragmatic role of FAD2–2 family and their exploitation in the biosynthesis of peakoil in soybean [17, 18].

The authors aimed to use CRISPR-Cas9 gene editing technology to mutate the FAD2–2 gene in soybean in order to improve the seed oil profile We generated transgenic soybean lines with the CRISPR transgene, tested for the presence of mutations in the FAD2–2 locus, assessed for off-target effects and determined impact on fatty acid profiles. Furthermore, we provide the current advancement and applications of CRISPR-Cas9 induced mutation driven by an oligo SgRNA sequence in plants may overcome major limitations of crop genetic improvement.

## Results

### CRISPR-Cas9 construction for target mutagenesis in *soybean*

**Fig. 1:**
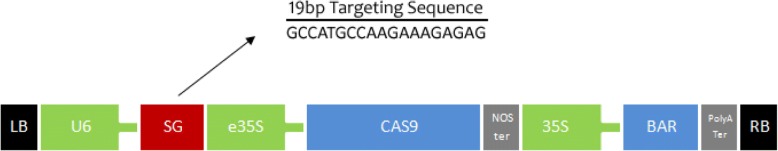
The schematic diagram of CRISPR-Cas9 binary vector (BGK041). This system includes 19 bp long Synthetic guide RNA (sgRNA), NOS terminator: NOS terminator is used for the termination signal of the gene expression, small U6 expression cassettes derived from soybean, CaMV 35S: promoter used to express the glufosinate resistant gene, BAR gene: A reporter herbicide resistance gene, LB: left border and RB: right border

**Fig. 2:**
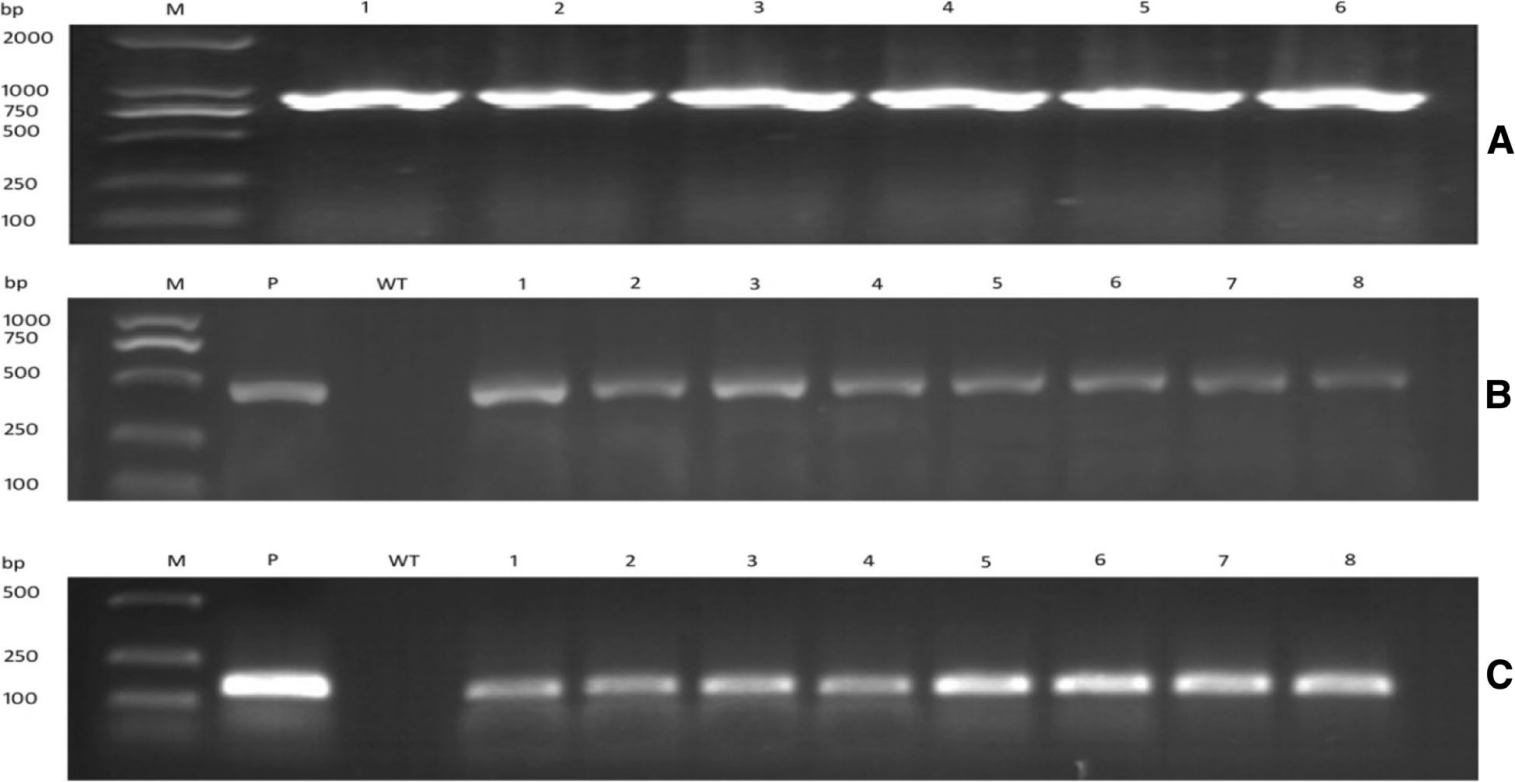
Detection of transgenic soybean plants. **a** Detection of CRISPR-Cas9 binary vector in Agrobacterium strain (EH105) using CRISPR specific primers where M: represents Marker or ladder and lanes 1–6 represents positive colons. **b** PCR analysis of BAR gene in transgenic plants of soybean, where wild type DNA (WT) and our constructed plasmid (P) were taken as negative and positive control. From 1 to 8 revealed different positive soybean plants. **c** Detection of NOS terminator in soybean transgenic plants. We used plasmid as positive control while (WT) represent negative, Lanes 1 to 8 indicates NOS terminator existence in soybean transformed plants. Based on these outcomes we confirmed the existence of the binary vector in soybean transgenic plants

We constructed a binary vector to improve the co-delivery of Cas9 gene and sgRNA in one expression vector CRISPR-Cas9 system (Fig. 1) under the e35S promoter for Cas9 gene and Soybean *U6* promoter which is important to drive sgRNA expression in plants [19]. Additionally, BAR gene was used in the same binary vector as a selectable marker gene which is derived from *Streptomyces hygroscopicu,* this contains modified phosphinothricin, which is solely responsible for herbicide resistance (glufosinate). *FAD2–2 microsomal omega-6 desaturase* gene of soybean was selected to identify targeted mutagenesis. The ligation of synthetic oligos (SgRNA) into CRISPR-Cas9 vector was performed according to manufacturer’s protocol. Positive clones of *Agrobacterium tumefaciens* (EHA105) carrying CRISPR-Cas9 were detected on YEP-kanamycin resistant medium. Further detection was confirmed through Polymerase chain reaction with G°Taq Flexi DNA polymerase (Promega Corp, Madison, WI) using CRISPR-Cas9 specific primers (Additional file 1: Table S1) (Fig. 2a).

### Detection of positive transgenic soybean lines

In our study, we used BAR gene as a selectable marker to detect the presence of transgene (SgRNA-CRISPR-Cas9) in transgenic soybean plants (Fig. 2b). Besides this, we also detected the presence of NOS terminator gene in target transgenic lines of soybean (Fig. 2c). For this purpose genomic DNA was extracted using (NuClean plant Genomic DNA Kit, Beijing, China) from the selected transgenic lines of soybean and subjected next to PCR amplification with G°Taq Flexi DNA polymerase (Promega Corp, Madison, WI) using forward and reverse pairs of primers for both NOS terminator and BAR gene. (Additional file 1: Table S2).

**Fig. 3:**
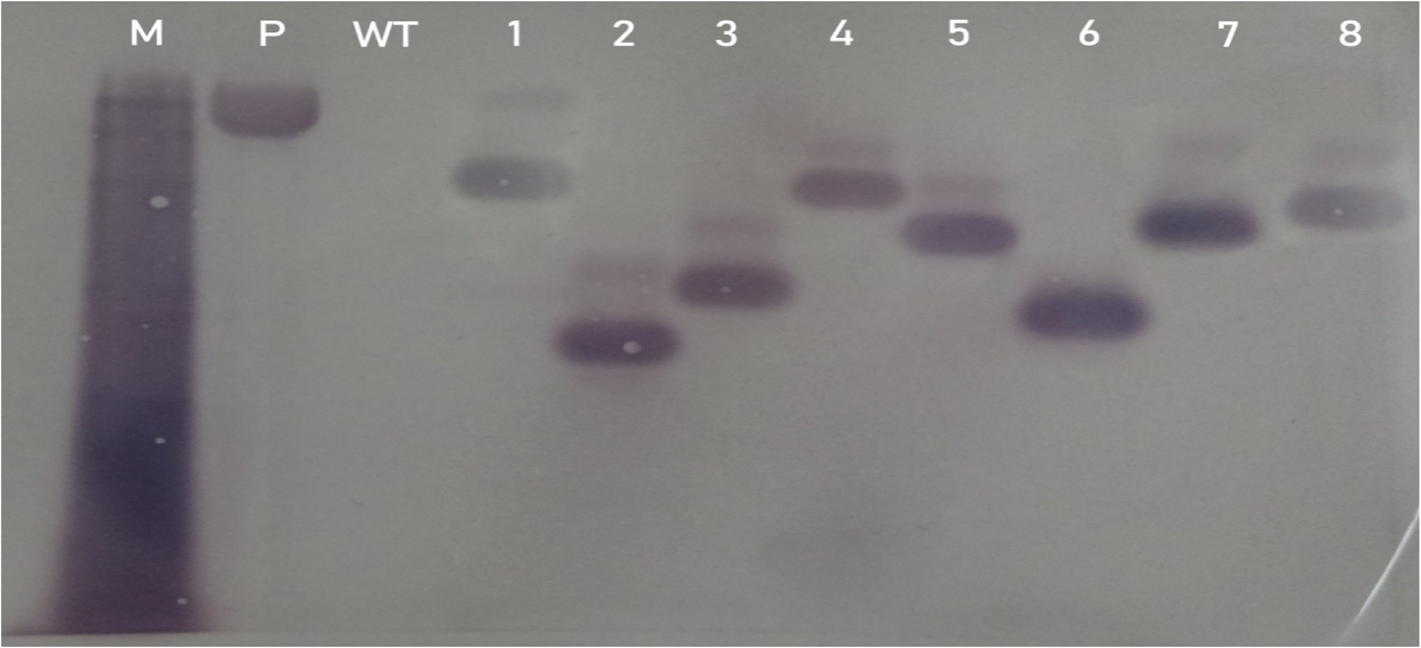
Southern blot hybridization of transgenic plants with BAR probe. The genomic DNA of 8 transgenic plants selected for detection followed by digestion of genomic DNA with restriction enzymes (HindIII and Tengo buffer). Where M represents marker, (P) positive control of plasmid, (W): Wild-type DNA, and from 1 to 8 demonstrate different transgenic plants

Consequently, further molecular detection of specific exogenous DNA (SgRNA-CRISPR-Cas9) copy number present in soybean mutant plants were analyzed by Southern blot analysis (Fig. 3).

A single enzyme digestion pattern with (HINDIII) enables the fractionation of BAR gene present in the transgenic soybean plants. Post hybridization all together 8 transformation events were detected with digoxigenin [DIG] 1-dUTP probe and DIG High Prime DNA Labeling chemicals separated on 0.8% agarose gel (Fig. 3). Various patterns of hybridization appear after immobilization of DNA onto nylon membranes (Amersham) showed a product of (552 bp) along with flanking sequence probes. This indicated the presence of our target exogenous DNA in several copies within mutant lines (Additional file 1: Figure S1).

### Detection of targeted mutation in transgenic soybean

**Fig. 4:**
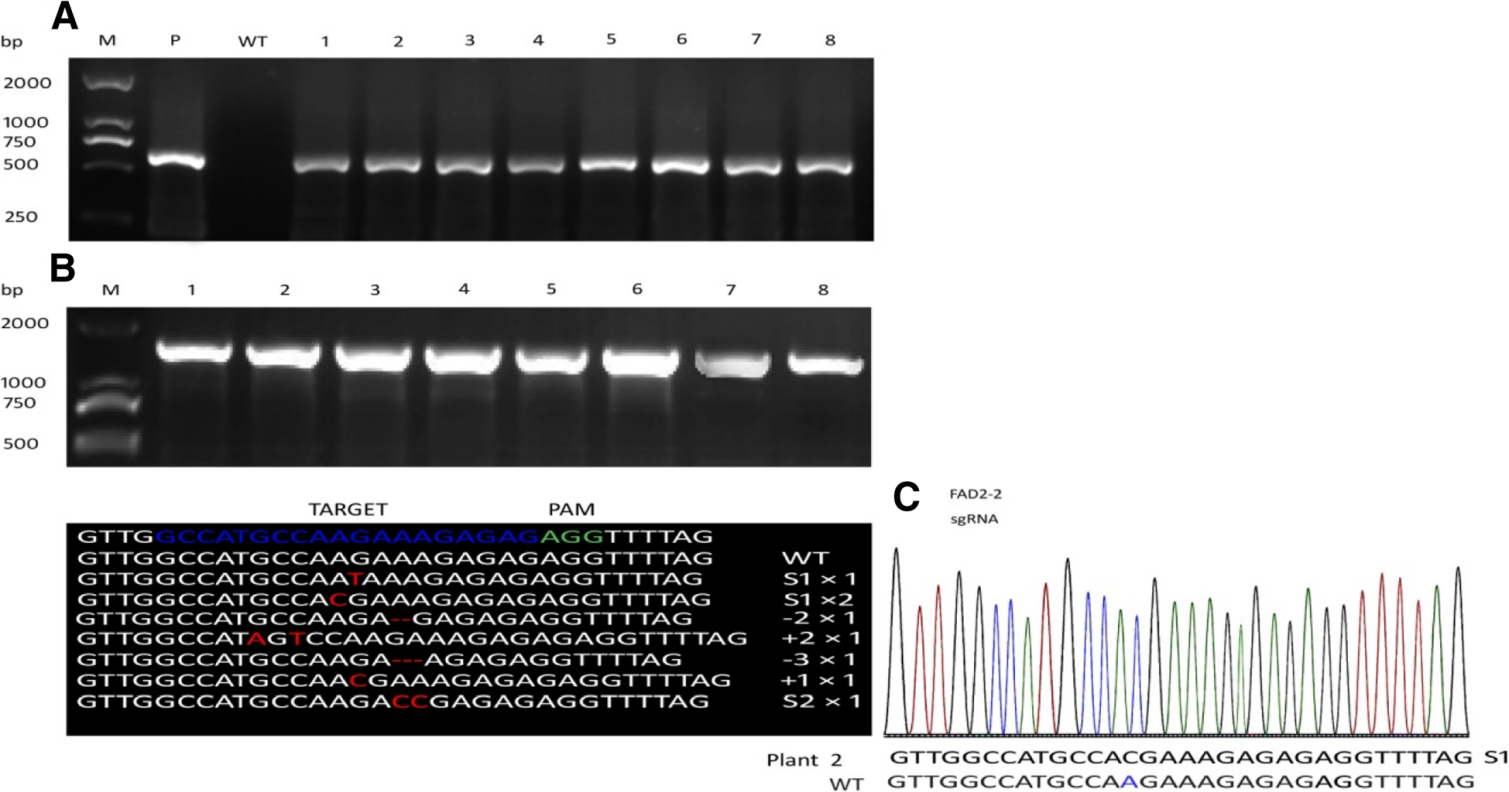
Detection of targeted mutation in transgenic soybean plants using CRISPR-Cas9 system. **a** PCR Detection of Cas9 gene in different soybean transgenic plants. We used plasmid DNA (P) as positive control and wild type DNA as negative (WT). The Series of 1 to 8 express different soybean transgenic plants. **b** The PCR product analysis of target gene (FAD2–2) of the independent transgenic plants, which is successfully cloned and subjected to sequence analysis for mutation detection. Sequences analysis revealed efficient targeted genome editing using CRISPR-Cas9 system. Red letters in the sequence indicate mutation where, S: indicate substitution, +: represent addition or insertion while/−: indicate deletion. The letters on right side show the number of transgenic plants where the mutations occurred in transgenic lines with the same type. . **c** Sequencing chromatograms of soybean transgenic plant. The blue color alphabets represent nucleotide substitution

Agrobacterium tumefacient mediated transformation has been reported as quick, most efficient, simple and low-cost method for the molecular study of plants. For the identification of the targeted mutagenesis, we established a binary vector system and then transformed it into soybean cotyledons via Agrobacterium mediated transformation. Transgenic plants were selected randomly to acquire the presence of Cas9 gene in soybean transgenic plants by PCR assay using Cas9 specific primers (Additional file 1: Table S2) (Fig. 4a). The target gene (FAD2–2) was also amplified and successfully cloned in T1 vector. The sequencing results verified for the presence of site-specific mutations at a specific locus. (338,046,125, 15:+ 22245082) (Fig. 4b). Following sequence metrics and chromatogram (Fig. 4c). We concluded that most of the mutations were found nucleotides substitutions while some were nucleotides insertion. Only in two sequences mutations were found as a deletion in the FAD2–2 gene locus (Fig. 4b, Additional file 1: Figure S3). The mutations rate was observed up to 21% in transgenic soybean plants (Table 1). However no biallelic mutations were detected according to the sequencing analysis (Fig. 4b).

**Table 1:** Mutation efficiency of FAD2–2 gene induced by CRISPR-Cas9 system

Target gene	No. of examined plant	No. of plants with cas9 gene	No. of plants with mutations	Mutation efficiency %
FAD2–2	556	37	8	21%

Mutation efficiency (%) = No of plants with mutations/No of plants with Cas9 gene×100

### Off-target analysis in soybean transgenic plants

CRISPR-Cas9 system is highly tolerated to numerous mismatches between the sgRNA and its target genes [20]. Additionally, it has high accuracy for targeted mutagenesis [21]. In the current study, we analyzed the potential off-target sites of FAD2–2-sgRNA2 in transgenic plants. After searching on CRISPR-P website, the potential off-target sequences of FAD2–2-sgRNA2 with PAM motif were identified (including *Glycine max* genome database. A total of 19 potential off-target sequences were analyzed. The off target mutation was further verified through PCR and sequence analysis (Additional file 1: Table S3). Transgenic soybean lines were analyzed for positive FAD2–2 (microsomal omega-6 desaturase) gene mutations and determine wherein both off-target and target mutations. After the complete assay of total 19 potentials off targets sequences (Additional file 1: Table S3) none of the potentials off target loci demonstrate CRISPR-Cas9 induced mutation (Table 2) Our findings suggested that CRISPR-Cas9 system had high specificity for the targeted mutagenesis in soybean plants.

**Table 2:** Analysis of targeted gene mutations induced by CRISPR-Cas9 cassettes in transgenic soybean lines. Transgenic plants containing confirmed FAD2–2 mutagenesis were subjected to look for off target editing

SgRNA	No. of putative off target sites	No. of loci of putative off target sites	No. of examined loci	No. of plants examined	No. of off target mutation
FAD2–2-sgRNA2	20	19	12	8	0

### qRT-PCR analysis

**Fig. 5:**
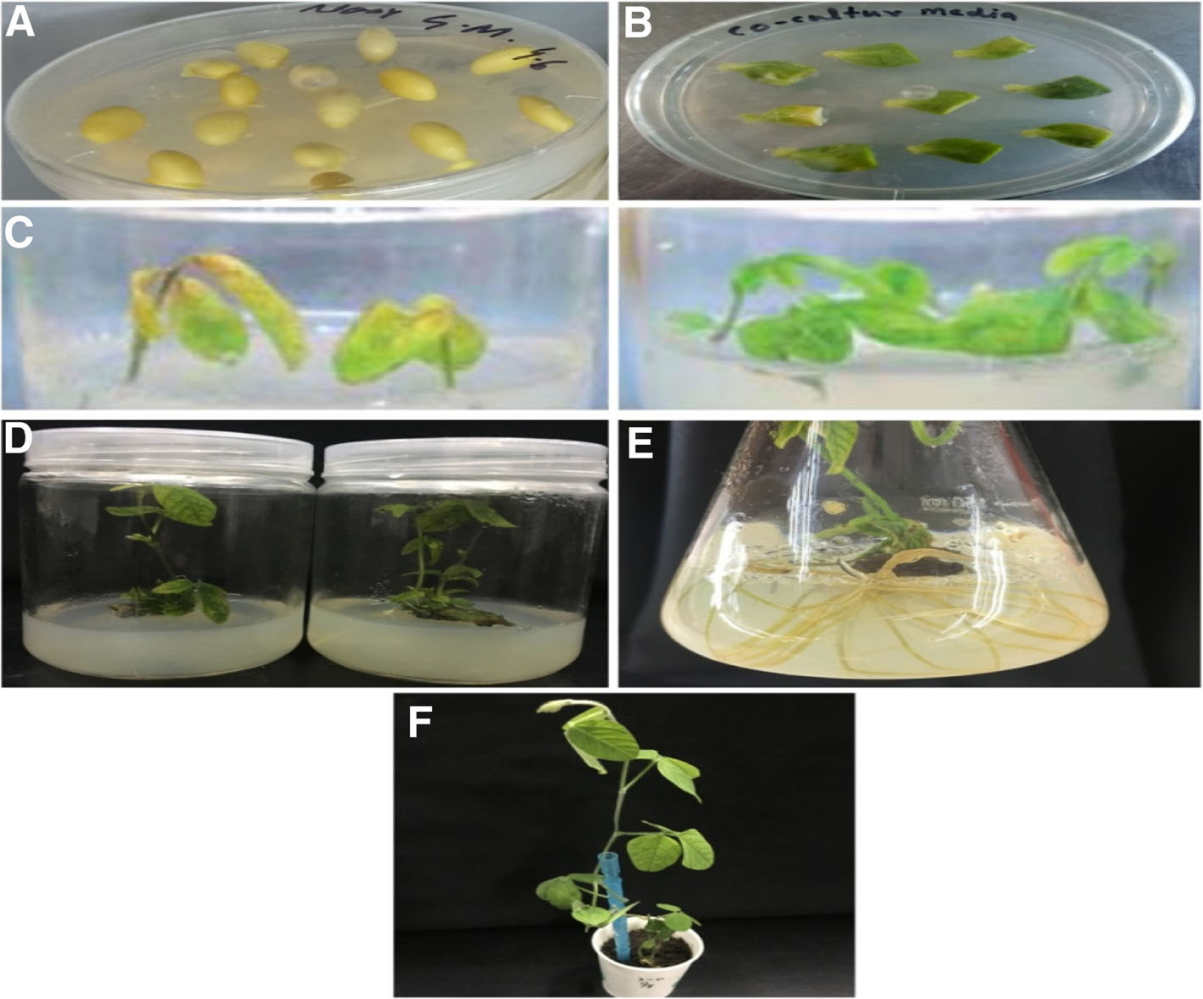
Tissue specific Quantitative real time expression analysis of CRISPR-Cas9 induced FAD2–2 gene. The relative expression level of mutant FAD2–2 gene in four different tissues of soybean including root, stem, leaf and seed. The abundance of mutant FAD2–2 transcripts was found in the leaf tissue of transgenic soybean lines. The data was normalized to that of *GmActin* gene (NM_001289231)

The relative expression ratio of a target gene was normalized with housekeeping gene (GmActin Gene, NM_001289231) and calculated according to the given equation r = E_target_ Δ^Cq target (calibrator-sample)/^E_reference_Δ^Cq reference (calibrator - sample)^. The promoter U6 is tissue-specific and temporally regulated and is not expressed in high frequency together in roots, stems, or leaves of soybean. However In this study, the gene FAD2–2 under promoter U6 relatively shows higher expression in leaf compared with seed, stem and root (Fig. 5).

### Fatty acid profiling via NIR

The measurements of fatty acid analysis of 8 selected transgenic soybean lines were carried out in order to conclude our expected results. We found drastic diversity in the increase/decrease of overall lipid contents of the mutant plants compared to wild soybean lines. The increased Oleic acid content was recorded up to (65.90%, P6) with the least level of linoleic acid (16.08%, P6). Moreover P8 oleic acid level (59.58%) with the low level of linoleic acid (19.05%) while in P7 (59.12) and (21.05%) respectively. These values depict significantly higher concentrations of oleic acid than the control (17.34%). Besides this, the level of palmitic acid decreased up to (6.95%, P6). These findings summarized the particular interest of the demand of high oleic acid content in soybean oil by manipulating FAD2–2 gene through a CRISPR-Cas9 system which simultaneously leads to low linoleic acid content in soybean oil (Table 3).

**Table 3:** The NIR analysis of complete fatty acid contents for FAD2–2 omega 6 destuarase mutants. Samples from P1-P8 represent the percentage composition of fatty acid contents in soybean transgenic lines

Plant	Palmitic	Stearic	Oleic	Linoleic	Linolenic
Control	10.49	2.75	17.34^h^ ± 0.20	59.54	8.92
P1	9.19	3.76	45.08^g^ ± 0.04	31.95	9.12
P2	8.76	2.94	51.21^e^ ± 0.11	27.10	8.64
P3	8.60	2.91	49.27^f^ ± 0.09	29.82	8.52
P4	8.19	2.90	58.82^c^ ± 0.37	20.10	8.63
P5	7.23	2.50	54.05^d^ ± 0.08	26.25	9.10
P6	6.95	2.86	65.90^a^ ± 0.33	16.08	7.12
P7	7.95	2.88	59.12^b^ ± 0.10	21.05	8.38
P8	8.35	2.82	59.58^b^ ± 0.22	19.05	8.90

## Discussion

Clustered regularly interspaced short palindromic repeat (CRISPR-Cas9). CRISPR-associated Cas9 nuclease can significantly induce targeted gene editing by cleaving target DNA sequence which is guided by synthetic sgRNA sequence incorporating double-strand break (DSB) on a target site [22]. Small guide RNA is the first 20 nucleotide sequences complementary to one strand of the target DNA with an NGG motif at 3′-end (the protospacer adjacent motif, PAM) which scans a target genome for editing. The expression of sgRNAs is carried by U3/U6 promoters, which transcribed sgRNA with the help of RNA polymerase [23, 24]. To induce targeted genome editing in vitro efficiently requires an efficient and feasible delivery of a binary vector consist of (Cas9 and sgRNA expression cassettes) into various plants. The inherited stability of CRISPR-Cas9 based mutations was reported previously [25]which can be transferred into next progeny. Here we have successfully demonstrated targeted genome editing in soybean using the CRISPR-Cas9 system, where most of the induced mutations were found as nucleotide substitution while some of them were nucleotide deletion and insertion (Fig. 4b). Our results were concordant with the previous findings [26]. The mutation efficiencies in transgenic plants were 21% (Table 1).

Fatty acid desaturases enzymes are contained in large numbers in plant. These enzymes are mainly present in endoplasmic reticulum and chloroplast. The fatty acid desaturases 2 (FAD2; EC 1.3.1.35) is hydroponic transmembrane endoplasmic reticulum protein, controlling the *cis* double bond between c12 and c13 [27]. The *FAD2–2* gene was initially reported in Arabidopsis [28], then it was further identified in several crops such as sesame (*Sesamum indicum*) [29], corn (*Zea mays*), canola (*Brassica napus*) [30], olive (*Olea europaea*) [31], soybean (*Glycine max*) [32], sunflower (*Helianthus annuus*) [33], and cotton (*Gossypium hirsutum*) [34]. Previously it has been stated that, the FAD2–2 gene is mainly responsible for the conversion of oleic acid into linoleic acid [29, 35]. Therefore, producing *FAD2–2* mutant could decrease the conversion of oleic acid into linoleic acid. Moreover, the *FAD2–2* gene expresses in all almost all plant tissues during oil biosynthesis but the expression level is relatively high in seeds [36]. Similarly, we observed high expression level of *FAD2-2* in stem and leaves comparing to seeds in both wild types (WT) and transgenic plant (T) (Fig. 5). However, there is a significant difference between the expression level of WT and T seeds tissue than that to other vegetative tissues, Where T plants showed relatively low expression level of *FAD2–2* gene in seeds. The result is in line with the previously reported study by Zhang et al. [37]. These finding further provide solace to the importance of *FAD2–2* genes family in the biosynthesis of oil from edible seed plants. For instance, the oleic acid content is considered as the most important and effective molecule in enhancing the nutritional index and shelf life of soybean oil. It also play role in decreasing the risk of type diabetes [38]. Though further study is required to produce double or triple mutant of *FAD2–2* genes in order to increase the value of oleic acid contents and restrict their conversion into linoleic acid. As it was suggested by Wang et al. in their study of *FAD2–2* genes family in peanut (*Arachis hypogaea* L.*)*[35]. In our findings, we found drastic diversity through NIR spectral data of overall lipid content of mutant plants seeds in comparison with wild soybean lines (Table 3). The maximum increase oleic acid content was recorded up to (65.90%, P6) while the lowest linoleic acid were found (16.08%, P6). These findings depict previous studies on FAD2–2 gene which encode ω-6 fatty acid desaturases catalyzes the conversion of oleic acid to linoleic acid in soybeans using antisense RNA technology [14, 39].

Additionally to tackle the most efficient and recent transformation method out of all available systems is a necessary step. In view of the fact that *Agrobacterium*-mediated transformation is proved the most widely used for potential applications of CRISPR-Cas9 which facilitate the efficient integration of T-DNAs consist of Cas9 and sgRNA expression cassettes in target plant [40]. We have successfully transferred a binary vector (pCas9-atU6-sgRNA) into soybean cotyledon via Agrobacterium tumafaciens (EH105). The transgenic lines were verified with a reporter gene (BAR), NOS terminator and Cas-9 gene (Figs. 2 and 4). Our study signifies the exploitation of FAD2–2 gene using CRISPR-Cas9 induced mutation in soybean which results in a significant increase oleic acid content (Table 3). Our results concluded that CRISPR-Cas9 system can play a vital role in constructing a mutant library of soybean.

## Conclusion

This work showed an efficient CRISPR-Cas9 gene editing technology to encounter FAD2–2 gene of soybean for targeted mutagenesis aimed to improve the fatty acid profiling of transgenic soybean lines. CRISPR transgene activity was detected by high throughput sequence analysis and southern blotting. The maximum induced rate of mutation was recorded 21% with high ratio of substitution. NIR analysis indicated the enhanced profiling of monounsaturated fatty acid content resulting in high oleic acid accumulation (65%).

## Materials and methods

### Experimental materials

Seeds of *Glycine max L*. (JN38) was obtained from previously stored stock in our lab (Plant biotechnology centre, Jilin Agricultural University, China). *Escherichia coli* strain (DH5α), Agrobacterium tumefacien strain (EHA105) and CRISPR-Cas9 vector was purchased from Biogle Co. Ltd. Hangzhou. Restriction enzymes, DNA extraction kit and DNA ligases were purchased from (Takara Biotechnology, Company Beijing). DIG High Prime DNA Labeling and Detection Starter kit 1 (for color detection with NBT/BCIP, REF 11745832910) were purchased from (Takara Biotechnology Company Beijing).

### Explants preparation

*Glycine max JN38* cultivar was selected for this study. After that, healthy seeds were selected and sterilized with 30 ml Cl_2,_ (25mlNaocl, 5 ml HCl) for 16 h, which was further used for Agrobacterium-mediated transformation.

### Vector construction

Prior to construction of our desired CRISPR Cas9 vector it is important to design our target synthetic sgRNA, for this purpose we extracted FASTA file of the complete Cds of (*FAD2–2 microsomal omega-6 desaturase*) gene having GenBank ACCESSION number (L43921) from NCBI. A BLASTN for (*FAD2–2 microsomal omega-6 desaturase*) sequence has been constructed against *Physcomitrella paten* in order to observe potential target sites within the coding regions. *Physcomitrella paten* is a model plant which is widely known for targeted gene knockouts and facilitating reverse genetics approaches.

The indication of percentage identity was recorded as 99.2% with best E. value in the exonic regions.. In line with previous studies a small guide RNA was designed at position 3:38046125, locus 15:+ 22245082 in the exonic region of *FAD2–2 gene.*

CRISPR-Cas9 (BGK041) plant gene knockout vector kit (Biogle Co. Ltd. Hangzhou, China) was used to construct the expression vector of the plant. The plasmid contain a codon-optimized *Cas9* gene under the e35S promoter, sgRNA gene with *U6* promoter from Soybean, NOS terminator for termination signal of the gene expression, and a reporter BAR gene (Fig. 1). The sgRNA scaffold (19-nt) target sequences were amplified by PCR using a pair of synthetic oligos according to manufacturers protocol (Additional file 1: Table S1). The annealing of oligos with CRISPR-Cas9 vector was followed according to manufacturer’s instructions and transformed into *E.coli strain* (DH5α).

### Preparation of agrobacterium tumafecian strain

*Agrobacterium tumefaciens* strain EHA105 stock was prepared. The transformation of EHA105 with CRISPR-Cas9 vector was accomplished with heat and cold shock method, followed by 45 mins shaking in LB liquid medium containing antibiotic (kanamycin 1 ml/L) and incubated overnight at 28 °C. The half colony was selected for PCR using CRISPR-Cas9 specific primers (Additional file 1: Table S2) (Fig. 2a) after selecting positive clones the remaining half was re-cultured for infection in 5 ml LB/1 ml kanamycin for overnight shaking at 28 °C, 220 rpm.

### Agrobacterium-mediated transformation of soybean

**Fig. 6:**
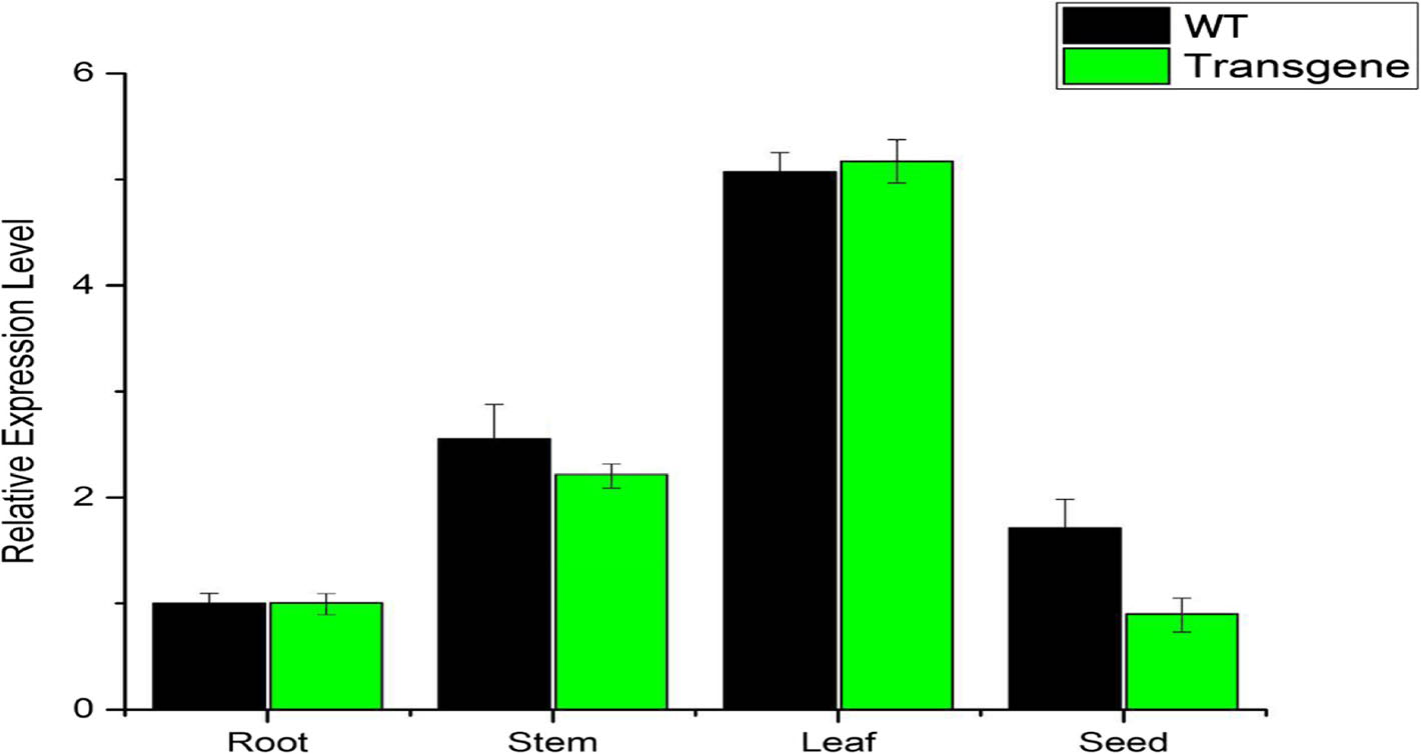
Agrobacterium mediated stable transformation of soybean. From A to F showed the series of different media to acquired stable transgenic soybean plants. **a** Sterilized soybean seeds were germinated on germination medium.**b** After infection with agrobacterium the seeds were planted in co-culture medium under controlled environment. **c** Collection of transgenic plants on a selective medium comprising 6 mg l^−1^glufosinate where left side medium indicates sensitivity while right side showed resistance to herbicide (glufosinate). **d** Glufosinate resistant seedlings were incubated on shoot elongation medium after 2 weeks. **e** Establishment of rootswere observed after 2 weeks of incubation on rooting medium. **f** A full flourish plant was shifted to sterile soil in a growth chamber

The cultivars JN38 (*Glycine max)* sterilized seeds were planted in germination media and placed in a full dark condition for 4–5 days (Fig. 6a). A half-seed method as described by Paz et al. [41] was used for A. *tumefacien* mediated transformation in soybean with minor modifications. The germinated seeds were taken and seed coat removal was done for easy bisection of seed then explants were prepared and converted to pre-culture media placed in full dark condition for a period of 3 days. Further, the explants (cotyledon node) were injured mechanically and infected with *A. tumefaciens* following 20 min self-shaking. The infected explants were re-suspended to co-cultivation media in dark place for 3 days (Fig. 6b) and then shifted to selective (S1) media and placed in a growth chamber under a controlled environment.

After 15 days incubation, the plants were observed and further transferred to selective (S2) media with herbicide treatment (glufosinate) for another period of 15 days (Fig. 6c). Finally, the explants which are glufosinate resistance were shifted to shooting media for 15 days (Fig. 6d) and then transferred to rooting media (Fig. 6e). Once root development was observed, double distilled water was added for 3 days, to make sure the damage less uprooting of plants. The uprooted plants further shifted to the sterilized soil for acclimatization (Fig. 6f, Additional file 1: Table S4).

### Progeny analysis of transgenic plants

We confirmed the presence of CRISPR-Cas9 using NOS terminator, BAR gene and Cas9 gene by PCR analysis with specific primers (Additional file 1: Table S3). Detection with BAR gene the PCR profile includes, Master mix 12.5 μl, DNA 2 μl, R/F primers 1 μl, dd water 8.5 μl making the total volume 25 μl and was set to a control of 35 cycles of 94 °C for 30s, 58 °C for 30 s, and 72 °C for 45 s. However the Cas9 gene utilizes follows the PCR system as, Master Mix 12.5 μl, DNA 2 μl, both primers 1 μl, dd water 8.5 μl making the total volume 25 μl and was set to a control of 35 cycles of 94 °C for 30s, 64 °C for 30 s, and 72 °C for 50 s. Each PCR products were examined on 1.2% agrose gel.

Genomic DNA of the transgenic plants was extracted using a NuClean plant Genomic DNA Kit (Beijing, China) following the company protocol. In order to identify the mutation in positive soybean plants, we amplified the target gene through Polymerase Chain Reaction with gene-specific primers (Additional file 1: Table S3). The PCR system was followed as, Master mix 12.5 μl, DNA 2 μl, primers 1 μl, dd water 8.5 μl making the total volume 25 μl and was set to a control of 35 cycles of 94 °C for 30s, 56 °C for 30 s, and 72 °C for 50s. The purification of selective target bands was obtained with Axygen DNA Gel Extraction Kit (Beijing, China) and then cloned into a pMD18-T vector for sequencing. Latterly the sequencing results were analyzed for mutation through software (DNA MAN). Several transgenic plants were selected and cloned for the detection of targeted mutation that has been acquired, (Additional file 1: Figure S2).

### Southern blot detection of transgenic plants

We selected positive plants for Southern blot analysis. Southern blot was performed with DIG High Prime DNA Labeling and Detection Starter kit 1 (for color detection with NBT/BCIP, REF 11745832910) with given manufacturer protocol. Genomic DNA was extracted from transgenic plants using a NuClean plant Genomic DNA Kit (beijing, China). DNA of transgenic plants was digested for overnight with hind111 and Tengo buffer and 1.2% gel was used for electrophoresis with a time interval of 2 h and then shakes in denatured solution for 2 h which was further placed in Amersham nylon membranes (Amersham) overnight. Marker BAR gene was extracted from transgenic DNA and used as a probe for detection. Afterward, 8 μl probe was mixed with 16 ml digoxigenin [DIG] 1-dUTP with DIG High Prime DNA Labeling chemicals. Hybridization was performed for 2 h at 42 °C and then further washed 2 times with 20 SSC. Furtherly, blocking and detection were performed according to the manufacturer’s protocol and then placed for detection at room temperature for 30 mints to 1 h.

### Off-target analysis

During the present study, we have analyzed the Potential off target of FAD2–2 on CRISPR-P website (http://cbi.hzau.edu.cn/cgi-bin/CRISPR) containing almost 49 plant genomes datasets including (*Glycine max* genomic database). The first 19 potential off-target sites were examined on CRISPR-P website, then we searched these 19 potential off-target sequences and their loci in *Glycine max* genomic database and identified with PCR and sequence analysis.

We also investigate various others factors affecting the optimum or minimal off target mutation in target genome. To avoid minimum off target mutations the selection of SgRNA within exonic region with low GC content can influence the effect of off target mutagenesis. The FAD2–2-sgRNA2 potential off target sites was identified and analyzed with sequencing analysis.

### Isolation of Total RNA and semi-quantitative RT-PCR analysis

The Total RNA was extracted using Trizol reagent (Invitrogen, Carlsbad, CA, USA) from different tissues of transgenic and wild type soybean plantlets. Further, purification of RNA was achieved with treatment of RNase-free DNaseI (TaKaRa, beijing, China) to eliminate genomic DNA contamination according to the protocols recommended by the manufacturer. The first strand of cDNA was synthesized from 2.0 μg of total RNA using the M-MLV First Strand Kit (Invitrogen) and the cDNA products equivalent to 200 ng of total RNA were used as templates in a 25 μL PCR reaction system. All conditions were followed according to previously described method [42]. Semi-quantitative RT-PCR analyses for gene expression were performed on a PCR instrument (S1000™ Thermal Cycler, BIO-RAD, Foster City, CA, USA), with the conditions of 95 °C for 30 s, followed by 40 cycles of 95 °C for 5 s and 60 °C for 30 s. The GmActin gene (NM_001289231) was used as a positive internal control and the gene primers for the qRT-PCR were designed using conserved sequences of FAD2–2 gene. (Additional file 1: Table S1).

### Fatty acid profiling via NIR

Soybean seeds were prepared for the near-infrared transmittance instrument (Infratec 1225) as described by [43]. The Calibration samples are taken from the soybean transgenic mutants. Fatty acid profiling consist of (Palmitic acid, stearic acid, oleic acid, linoleic and linolenic acid) were selected for the prediction of NIRs in soybean seed. The results were significantly analyzed with statistical analysis by calculating their mean values.

### Bioinformatics analysis

The complete CDs of *Glycine max FAD2–2 microsomal omega-6 desaturase* in FASTA format were extracted from GenBank (L43921). This 1556 bp long fragment is then subjected to BLASTN against *Physcomitrella patens* genome using ENSEMBL PLANTS. To accomplish the potential Targeted sites for sgRNA within FAD2–2 sequence the filtered sequence is further subjected to CRISPR-P, and then selected the target genome of FAD2–2 (*Glycine max L.)* for which we were intended to design a highly specific targeted sgRNA sequence.

## Additional file

Additional file 1: **Table S1.** List of primers used in this study. Synthetic sgRNA/oligos 2. CRISPR vector primers sequences 3.qRT-PCR Primers sequences. **Table S2.** List of primers for mutation detection. BAR gene primers sequences b. NOS terminator primers sequences c. Cas9 gene primers sequences. **Table S3.** Potential off-target sites identified for *FAD2–2* target sequence in (*Glycine max*) genome. Potential off target sites were tested for FAD2–2 target sequence in soybean genome where red color indicates mismatching bases. **Figure S1.** Gels and Blots. A. Detection of CRISPR-Cas9 binary vector in Agrobacterium strain (EH105) using CRISPR specific primers (900 bp). B. Detection of NOS terminator (192 bp) and BAR gene (552 bp) in transgenic soybean. C. Southern blot of transgenic plants with BAR probe. D. Detection of Cas9 gene (663 bp) in different soybean transgenic plants. E. The PCR product analysis of target gene FAD2–2 (1556 bp) of the independent transgenic plants. **Figure S2. **Transgenic soybean plants mediated by Agrobacterium tumafecians. Strongest transgenic soybean plants after acclimatization. **Table S4.** Different media and its composition for *Agrobacterium* mediated transformation of soybean (JN38). Chemical composition of germination medium, pre-culture medium, infection medium, co-culture medium, selective medium1, selective medium2, elongation medium and rooting medium. **Figure S3.** List of Chromatograms obtained in our study. 1): (+ 1) represents addition of one nucleotide. 2): (+ 2) represents addition of two nucleotides. 3: (− 2) indicates deletion of two nucleotides. 4):(− 3) indicates deletion of three nucleotides. 5):(S1) represents substitution of one nucleotide. 6):(S2) represents substitution of two nucleotides. (DOCX 1270 kb)

## Abbreviations

CDs: Coding sequences
CRISPR: Clustered regularly interspaced short palindromic repeat
DIG: Digoxigenin
NIR: Near-infrared spectroscopy
PAM: Protospacer adjacent motif
SgRNA: Single guide RNA
TALENs: Transcription Activator-Like Effectors Nucleases
ZFNs: Zinc Finger Nucleases
