# Correction to: CRISPR-Cas9 mediated targeted disruption of FAD2–2 microsomal omega-6 desaturase in soybean (Glycine max.L)

**DOI:** 10.1186/s12896-020-00634-x

**Published:** 2020-08-20

**Authors:** Noor al Amin, Naveed Ahmad, Nan Wu, Xiumin Pu, Tong Ma, Yeyao Du, Xiaoxue Bo, Nan Wang, Rahat Sharif, Piwu Wang

**Affiliations:** 1 College of Agronomy, Plant Biotechnology Center, Jilin Agricultural University, Changchun, 130118 Jilin China; 2 Ministry of Education Engineering Research Center of Bioreactor and Pharmaceutical, Development Jilin Agricultural University, Changchun, 130118 Jilin China

**Correction to: BMC Biotechnol 19, 9 (2019)**

**https://doi.org/10.1186/s12896-019-0501-2**

Following publication of the original article [1], the authors identified that Figs. 5 and 6 were interchanged. There are also some other small mistakes identified. The correct figures and captions have been included in this correction. Other textual corrections are given below and have been highlighted in **bold typeface**.

**Fig. 5:**
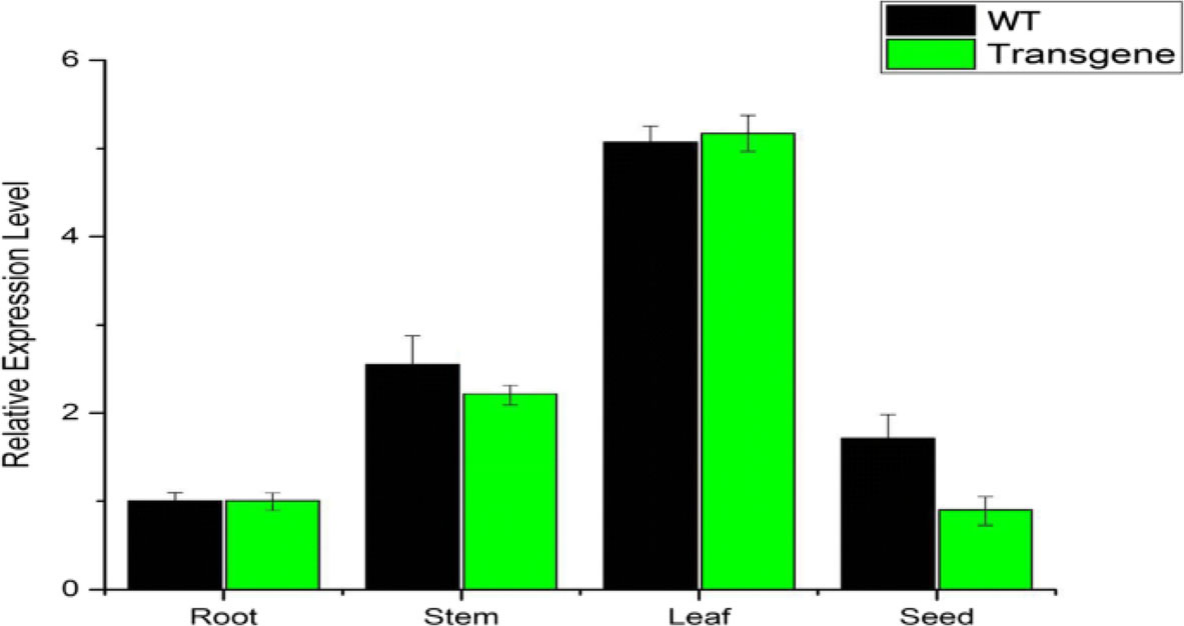
Tissue specific Quantitative real time expression analysis of CRISPR-Cas9 induced FAD2–2 gene. The relative expression level of mutant FAD2–2 gene in four different tissues of soybean including root, stem, leaf and seed. The abundance of mutant FAD2–2 transcripts was found in the leaf tissue of transgenic soybean lines. The data was normalized to that of *GmActin* gene (NM_001289231)

**Fig. 6:**
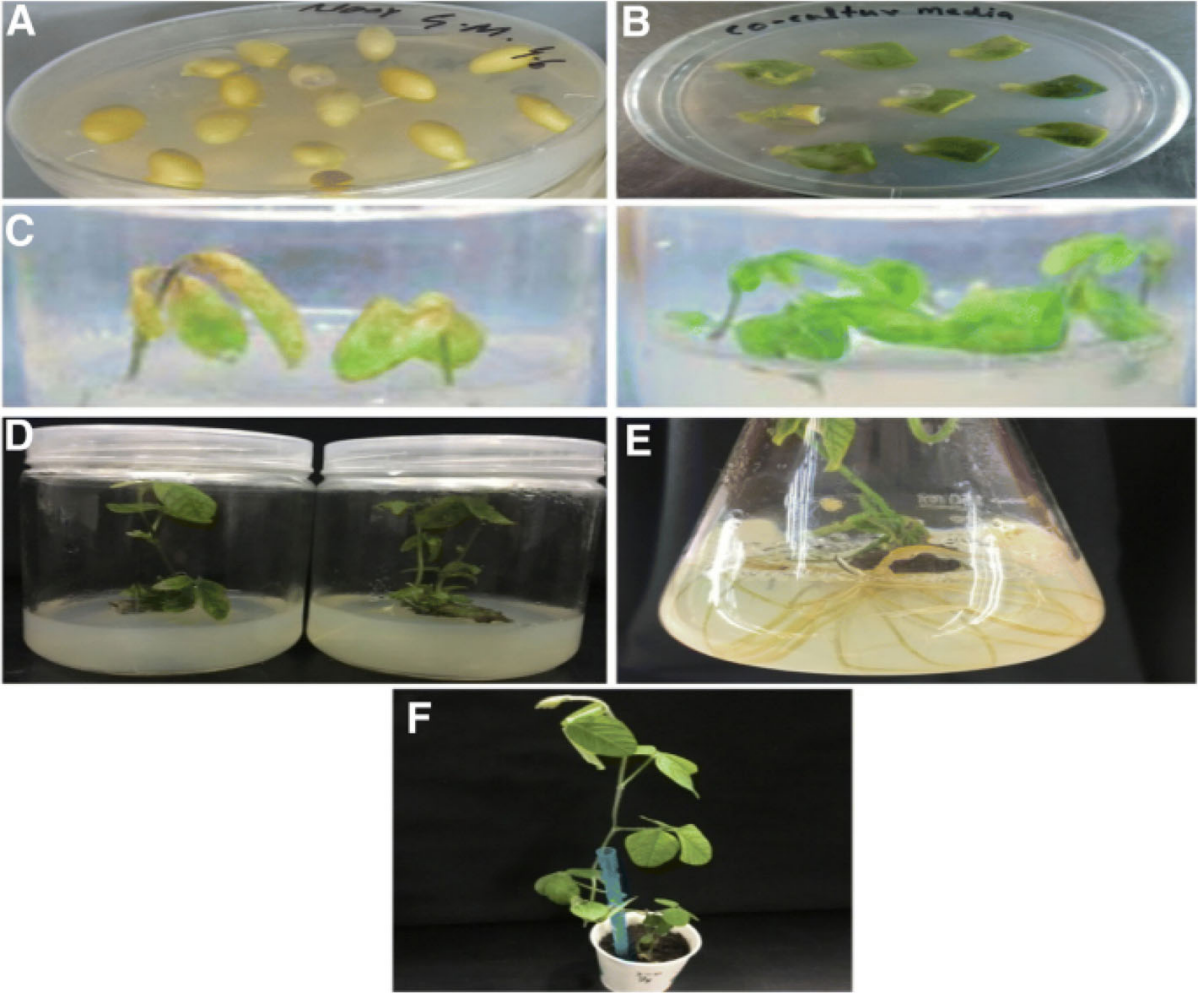
Agrobacterium mediated stable transformation of soybean. From A to F showed the series of different media to acquired stable transgenic soybean plants. **a** Sterilized soybean seeds were germinated on germination medium. **b** After infection with agrobacterium the seeds were planted in co-culture medium under controlled environment. **c** Collection of transgenic plants on a selective medium comprising 6 mg l^−1^glufosinate where left side medium indicates sensitivity while right side showed resistance to herbicide (glufosinate). **d** Glufosinate resistant seedlings were incubated on shoot elongation medium after 2 weeks. **e** Establishment of rootswere observed after 2 weeks of incubation on rooting medium. **f** A full flourish plant was shifted to sterile soil in a growth chamber

The correct figure and legends for Figs. 5 and 6 are:

**In addition, the following textual errors were noted and corrected text is indicated below:**

1. ‘Hind111’ in ‘Southern blot detection of transgenic plants’ is HindIII
2. The enzyme name ‘microsomal-6 desaturase’ is ‘microsomal omega-6 desaturase’.
3. The word ‘transgene’ in the key of Fig. 5 should read ‘Transgenic lines’.
4. ‘G°Taq Flexi DNA polymerase’ within the results ‘CRISPR-Cas9 construction for target mutagenesis in *soybean’* is ‘Go Taq Flexi DNA polymerase’.
5. ‘Tengo’ buffer stated in Fig. 3 legend is corrected to ‘Tango’ buffer.
