# Supplementary material for: CRISPR-Cas9 mediated targeted disruption of FAD2–2 microsomal omega-6 desaturase in soybean (Glycine max.L)
Source: BMC Biotechnol. 2019 Jan 28;19:9. doi: 10.1186/s12896-019-0501-2 (PMC6350355; doi:10.1186/s12896-019-0501-2)
Supplement: Supplementary file 1 — Table S1. List of primers used in this study. Synthetic sgRNA/oligos 2. CRISPR vector primers sequences 3.qRT-PCR Primers sequences. Table S2. List of primers for mutation detection. BAR gene primers sequences b. NOS terminator primers sequences c. Cas9 gene primers sequences. Table S3. Potential off-target sites identified for FAD2–2 target sequence in (Glycine max) genome. Potential off target sites were tested for FAD2–2 target sequence in soybean genome where red color indicates mismatching bases. Figure S1. Gels and Blots. A. Detection of CRISPR-Cas9 binary vector in Agrobacterium strain (EH105) using CRISPR specific primers (900 bp). B. Detection of NOS terminator (192 bp) and BAR gene (552 bp) in transgenic soybean. C. Southern blot of transgenic plants with BAR probe. D. Detection of Cas9 gene (663 bp) in different soybean transgenic plants. E. The PCR product analysis of target gene FAD2–2 (1556 bp) of the independent transgenic plants. Figure S2. Transgenic soybean plants mediated by Agrobacterium tumafecians. Strongest transgenic soybean plants after acclimatization. Table S4. Different media and its composition for Agrobacterium mediated transformation of soybean (JN38). Chemical composition of germination medium, pre-culture medium, infection medium, co-culture medium, selective medium1, selective medium2, elongation medium and rooting medium. Figure S3. List of Chromatograms obtained in our study. 1): (+ 1) represents addition of one nucleotide. 2): (+ 2) represents addition of two nucleotides. 3: (− 2) indicates deletion of two nucleotides. 4):(− 3) indicates deletion of three nucleotides. 5):(S1) represents substitution of one nucleotide. 6):(S2) represents substitution of two nucleotides. (DOCX 1270 kb) [file 12896_2019_501_MOESM1_ESM.docx]

**CRISPR-Cas9 mediated targeted disruption of *FAD2-2 microsomal omega-6 desaturase*** **in soybean (*Glycine max.L*).**

**Authors' information list:**

**Nooral Amin: PhD Scholar**

**Naveed Ahmad:** **PhD scholar**

**Wu Nan : PhD scholar**

**Pu Xiumin: Master Scholar**

**Ma Tong: Master Scholar**

**Duyeyao: PhD scholar**

**Bo Xiaoxue: Master Scholar**

**Wang Nan: Master Scholar**

**Rahat sharif : Master Scholar**

**Wang Piwu: Professor**

**^1^**College of Agronomy, Plant Biotechnology Center, Jilin Agricultural University, Changchun 130118, Jilin, China

**^2^**Ministry of Education Engineering Research Center of Bioreactor and Pharmaceutical Development Jilin Agricultural University, Changchun 130118, Jilin, China

**Table S1 List of primers used in our study.**

| **1.Synthetic sgRNA/ oligos** | **2.CRISPR vector primers** | **3.qRT-PCR Primers** |
| --- | --- | --- |
| **Oligo up (F):** GGGTTGCCATGCCAAGAAAGAGAGA  **Oligo low (R):**AAACTCTCTCTTTCTTGGCATGGCA | **CRISPR-F:** GCAGGCCCTGTGCAGGTAAG  **CRISPR-R:** CAGGAAACAGCTATGAC | **qRT-F :** TCACCACTCCAACACTGGTTC  **qRT-R:** AGGGAAAGTTCAAAGAAGCCT |

**Table S2 List of primers for mutation detection.**

| 1. **BAR gene primers** | 1. **TNOS primers** | 1. **Cas9 gene primers** |
| --- | --- | --- |
| BAR-F: TCAAATCTCGGTGACGGGC  BAR-R: GTCTGCACCATCGTCAACCACTA | NOS-F:GAATCCTGTTGCCGGTCTTG  NOS-R: TTATCCTAGTTTGCGCGCTA | Cas9-F: CCCAAGAGGAACAGCGATAAG  Cas9-R: GTCGATGGTGGTGTCAAAGT |

**Table S3** **Potential off-target sites identified for *FAD2-2* target sequence in (Glycine max) genome.**

| Sequence of off target sites | No. of maching bases and mismaching include with PAM | Off target locus | Tested? |
| --- | --- | --- | --- |
| GCCATGCCAAGAAAGAGA**A**A**GG** | (1MMs) 21 | 10:+25937632 | Yes |
| GCCATGCCAAGAAAGAGA**A**A**GG** | (1MMs) 21 | 6:-41343433 | Yes |
| GCCATGCCAAGAAAGAGA**A**A**GG** | (1MMs) 21 | 9:-16319096 | Yes |
| GC**A**ATG**A**CAAGAAAGAGAGA**GG** | (2MMs) 20 | 3:-38033709 | Yes |
| GC**TT**TGCCAAGAAAGAGAGA**T**G | (3MMs) 19 | 10:+12614307 | No |
| G**A**C**T**TG**A**CAA**A**AAAGAGAGA**AG** | (4MMs) 18 | 11:-7869787 | No |
| G**A**CATGC**A**AAGA**G**AGAGAGA**GG** | (3MMs) 19 | 3:+39125685 | Yes |
| GC**T**ATGC**A**AAGA**G**AGA**A**AGA**GG** | (4MMs) 18 | 8:-27157908 | Yes |
| G**AA**ATGCCAAGAAAGA**A**A**T**A**AG** | (4MMs) 18 | 9:-35125365 | Yes |
| GC**T**AT**A**C**T**AAGAAA**A**AGAGA**GG** | (4MMs) 18 | 15:-21762966 | Yes |
| G**A**CATGCCAAGAAAGAGA**C**A**A**G | (3MMs) 19 | 16:+5580184 | No |
| G**A**C**T**TGCCAAGAAAGAGA**T**A**T**G | (4MMs) 18 | 11:+5012102 | No |
| G**GG**ATG**AA**AAGAAAGAGAGA**GG** | (4MMs) 18 | 15:-36827008 | No |
| G**GG**ATG**AA**AAGAAAGAGAGA**AG** | (4MMs) 18 | 11:-12723958 | No |
| G**GG**ATG**AA**AAGAAAGAGAGA**AG** | (4MMs) 18 | 12:-13484290 | Yes |
| G**A**CATGCCA**T**GA**G**AGAGAGA**GG** | (3MMs) 19 | 4:-18777604 | Yes |
| G**A**CATGC**A**AA**T**AAAGAGA**A**A**AG** | (4MMs) 18 | 18:-50434884 | Yes |
| GCCATGCCAA**A**AAAG**GA**A**A**A**CG** | (4MMs) 18 | 15:-25592567 | Yes |
| GCCATG**A**CA**T**GA**G**AGAGAGA**GG** | (3MMs) 19 | 8:+44068919 | Yes |

Red color represents mismatching bases.

**Figure S1 Gels and Blots.**
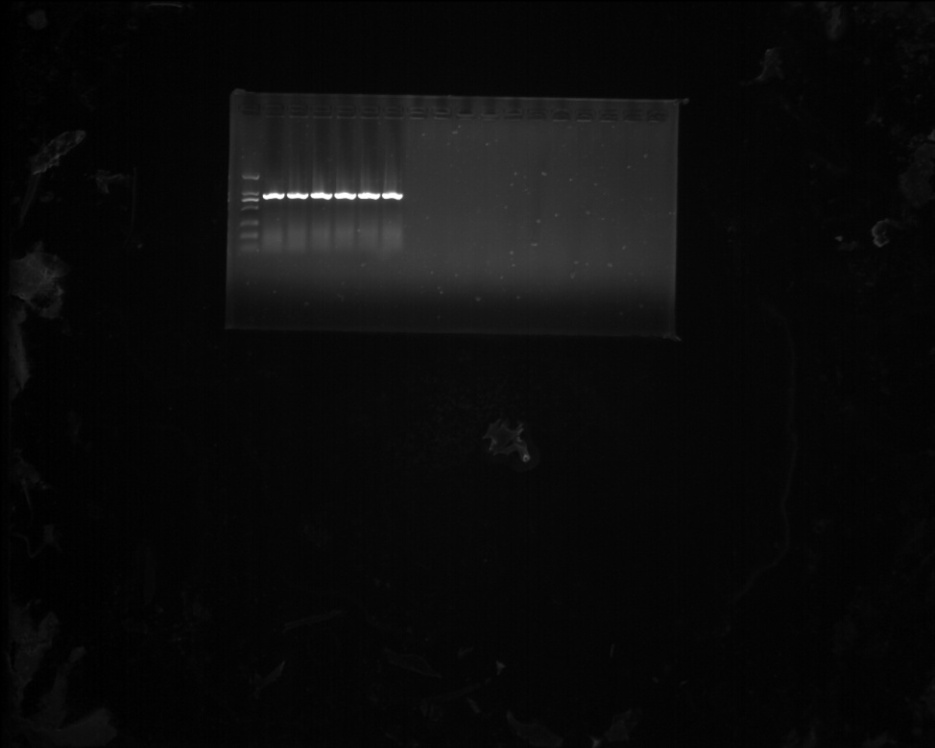
**A. Detection of CRISPR-Cas9 binary vector in Agrobacterium strain (EH105) using CRISPR specific primers.(900 bp)**

**B. Detection of NOS terminator (192 bp) and BAR gene (552 bp) in transgenic soybean.**

**
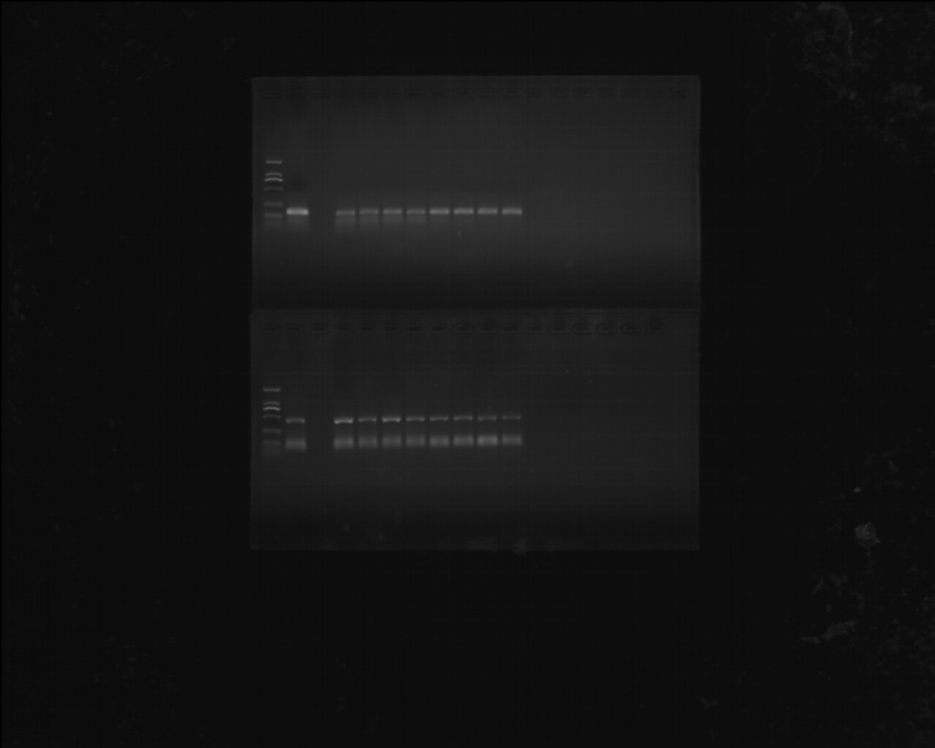
**

**C. Southern blot of transgenic plants with BAR probe.**

**
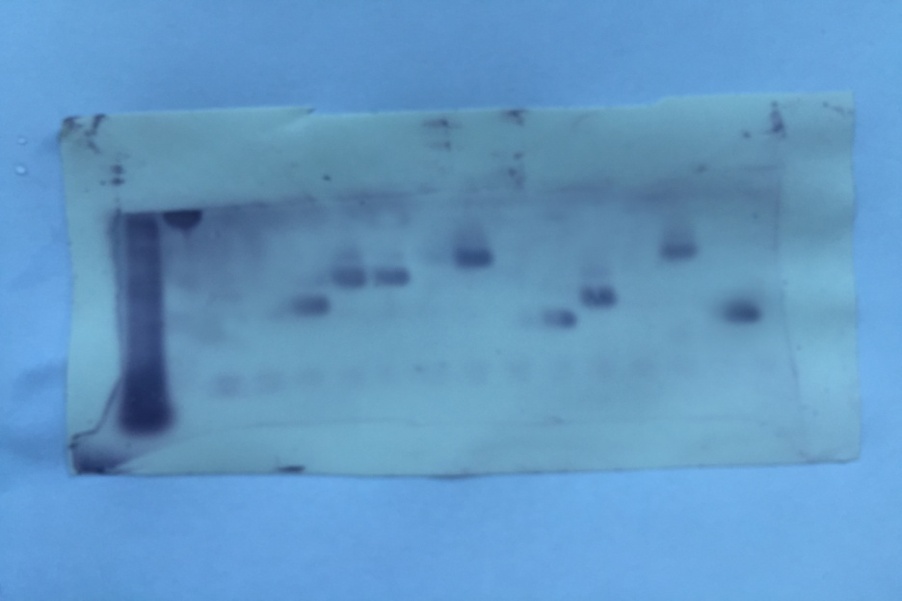
**

**
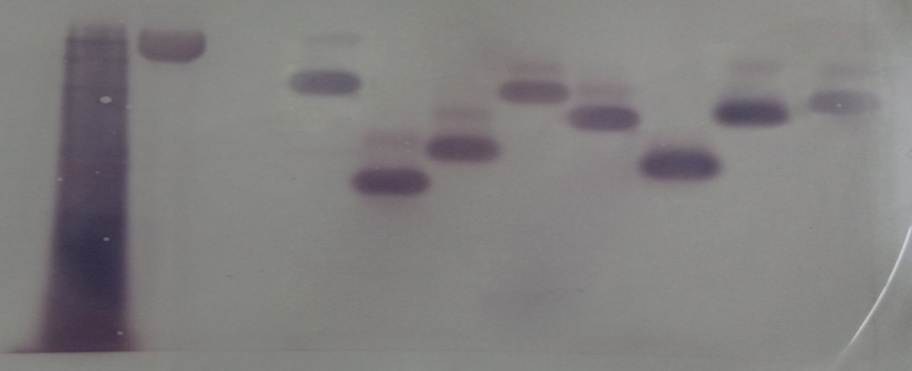
**

**D. Detection of Cas9 gene (663 bp) in different soybean transgenic plants.**

**
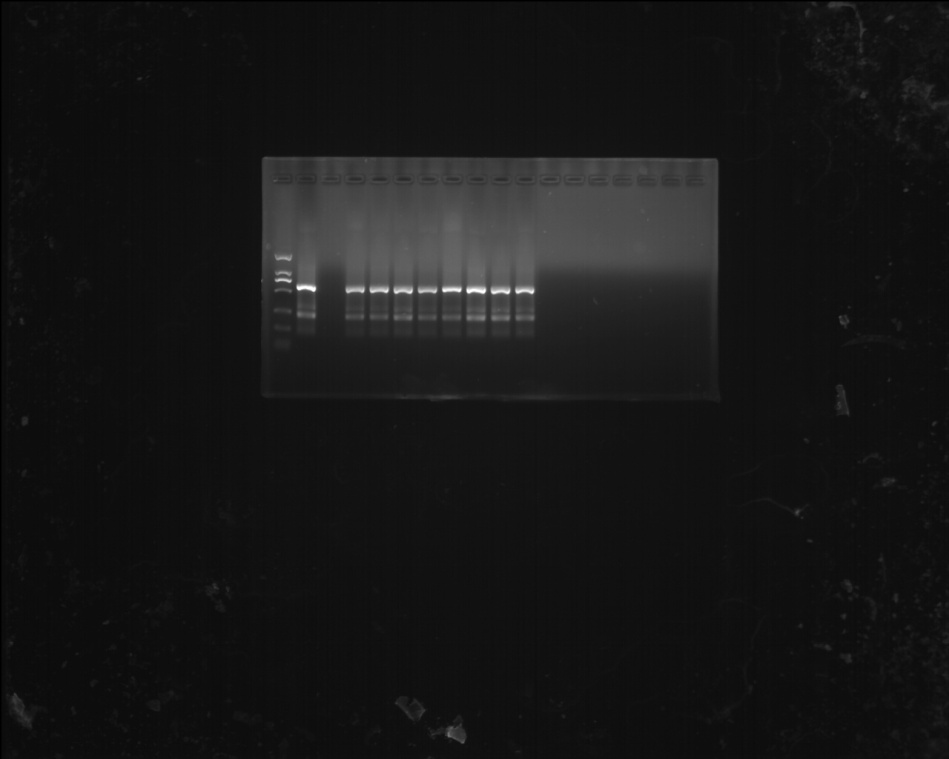
**

**F**

**E. The PCR product analysis of target gene FAD2-2 (1556 bp) of the independent transgenic plants**

**
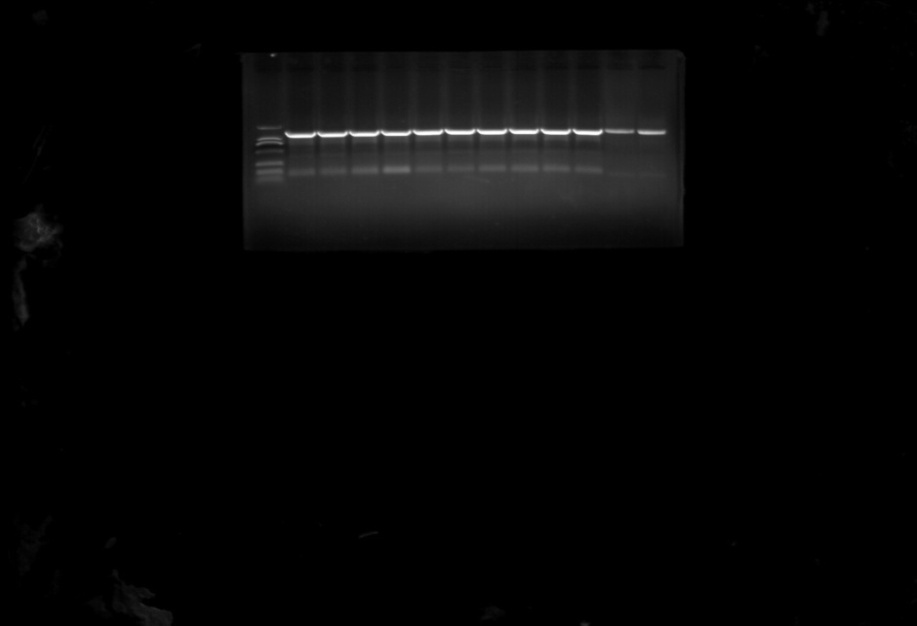
**

**Figure S2 Transgenic soybean plants mediated by *Agrobacterium tumafecians*.**

**
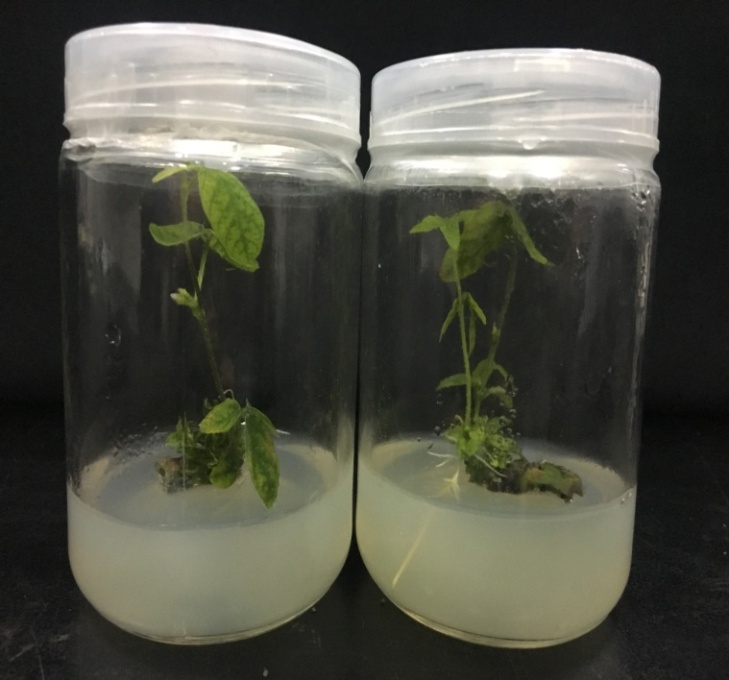

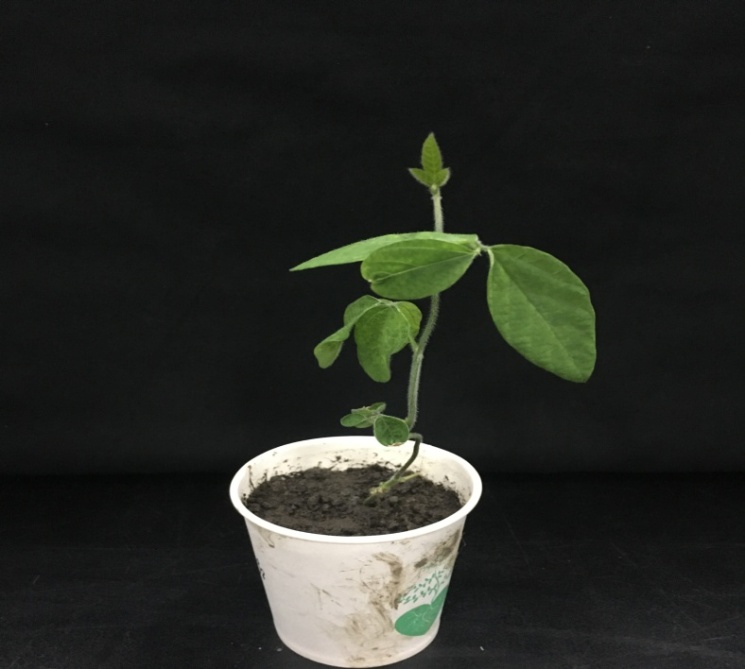
**

**
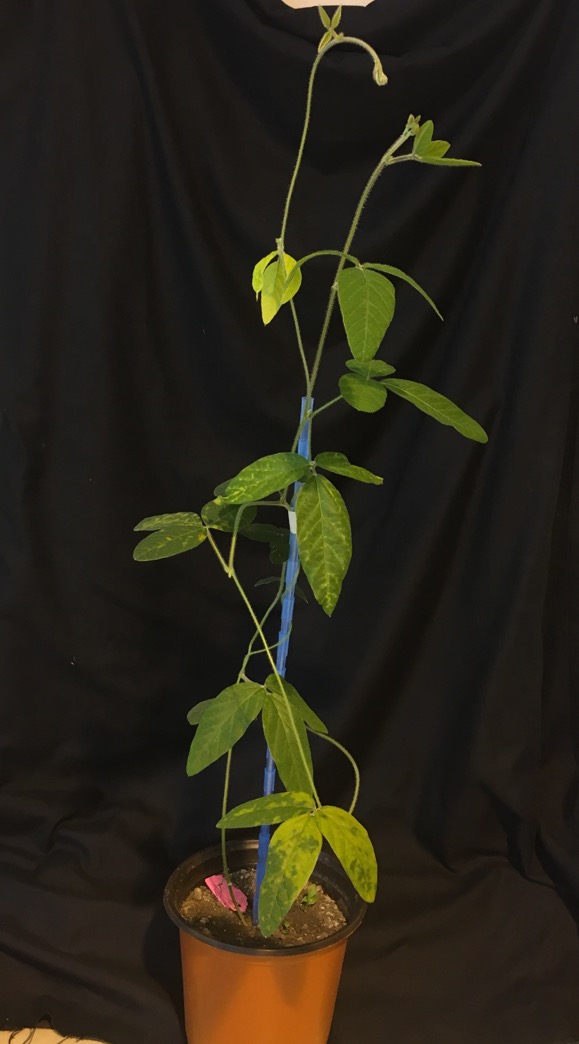
**

**Table S4 Different media and its composition for agrobacterium mediated transformation of soybean (JN38).**

| Media code | Composition |
| --- | --- |
| Germination media | MS macronutrients 5ml/L, MS trace 1ml/L, B5 organic 1ml/L, /ferric salt 1ml/L, MES 0.059g/L, sucrose 3g/L, agar 7g/L,  (PH5.80-5.86). The beaker was placed on shaker and autoclaved. |
| Pre-culture media | MS macronutrients 50ml/L, MS trace 10ml/L, B5 organic 10ml/L, ferric salt 10ml/L, MES 0.59g/L, sucrose 30g/L, agar 6.5g/L, (PH5.80). Filter sterilized 6BA 1g/L and IBA 1gl/L was added after autoclaving. |
| infect media | MS macronutrients 50ml/L, MS trace 10ml/L, B5 organic 10ml/L, ferric salt 10ml/L, MES 0.59g/L, sucrose 30g/L, (PH5.80). Filter sterilized 6BA 1g/L, IBA 1g/L and AS 19.26/L, was added after autoclaving. |
| Co-culture media | MS macronutrients 50ml/L, MS trace 10ml/L, B5 organic 10ml/L, ferric salt 10ml/L, MES 0.59g/L, sucrose 30g/L, agar 6.5g/L, (PH5.80-5.86). placed on shaker for shaking and then Filter sterilized 6BA 1g/L, IBA 1g/L and AS 19.26g/L, were added after autoclaving. |
| Selective media 1 | MS macronutrients 50ml/L, MS trace 10ml/L, B5 organic 10ml/L, ferric salt 10ml/L, MES 0.59g/L, sucrose 30g/L, agar 6.5g/L, (PH5.80-5.86). Filter sterilized 6BA 1g/L, IBA 1g/L, Carb 100g/L, and Cef 250mg/L were added after autoclaved. |
| Selective media 2 | Same as selective medium I, in addition with basta(glufosinate) 5mg/L, solidified with 6.80 agar at pH 5.8. |
| Elongation media | MS macronutrients 50ml/L, MS trace 10ml/L, B5 organic 10ml/L, ferric salt 10ml/L, MES 0.59g/L, sucrose 30g/L, agar 6.5g/L, (PH5.80-5.86). Filter sterilized IBA 1g/L, Carb 100g/L, Cef 250mg/L and GA 1g/L were added after autoclaved. |
| Rooting media | MS macronutrients 50ml/L, MS trace 10ml/L, B5 organic 10ml/L, ferric salt 10ml/L, MES 0.59g/L, sucrose 30g/L, agar 6.5g/L, (PH5.80-5.86). Filter sterilized IBA 1g/L, Carb 100g/L, and Cef 250mg/L were added after autoclaved |

**Figure S3. List of Chromatograms obtained in our study.**


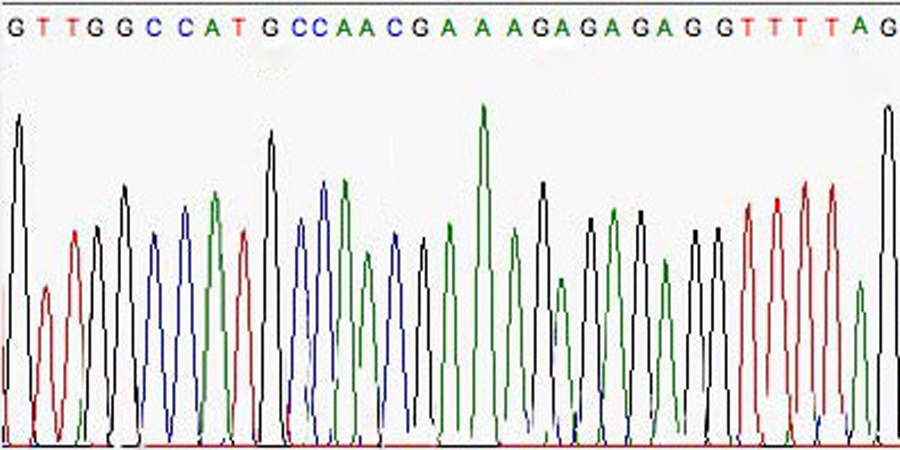
1): (+1)


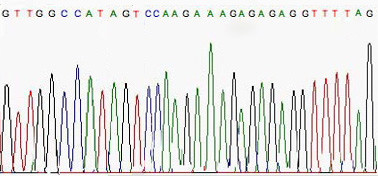
2): (+2)


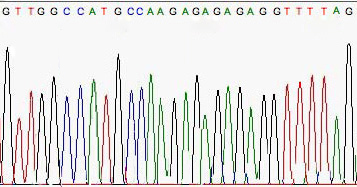
3): (-2)

4):(-3)


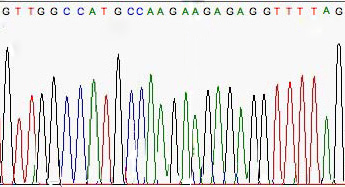


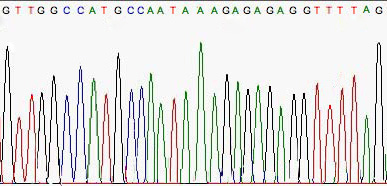
5):(S1)


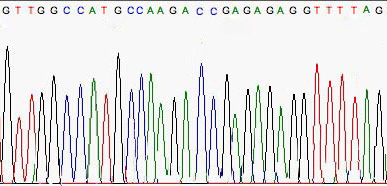
6):(S2)
